# Research landscape and emerging themes of ferroptosis in sepsis-associated acute kidney injury: a bibliometric and science-mapping analysis (2016–2025)

**DOI:** 10.3389/fphar.2026.1942452

**Published:** 2026-09-24

**Authors:** Junhu Li, Shuqin Tang, Shasha Tian, Dongyun Wang, Zhijuan Wu, Zheyu Yue, Zhibo Zhao, Xiaoshuang Zhou

**Affiliations:** 1 Department of Nephrology, Shanxi Provincial People’s Hospital, The Fifth Clinical Medical College of Shanxi Medical University, Taiyuan, China; 2 Big Data Center of Kidney Disease, Shanxi Provincial People’s Hospital, Taiyuan, China; 3 Shanxi Provincial Key Laboratory of Kidney Disease, Taiyuan, China; 4 Medicinal Basic Research Innovation Center of Chronic Kidney Disease, Ministry of Education, Shanxi Medical University, Taiyuan, China; 5 Department of Nephrology, Dazhou Central Hospital, DaZhou, China; 6 Emergency Management Office of Shanxi Provincial People’s Hospital, Taiyuan, China; 7 CollegeIII of Clinical Medicine, Shanxi University of Traditional Chinese Medicine, Taiyuan, China

**Keywords:** bibliometric analysis, co-citation analysis, ferroptosis, science mapping, sepsis-associated acute kidney injury

## Abstract

**Background:**

Ferroptosis has received increasing attention as a regulated cell-death process potentially involved in sepsis-associated acute kidney injury (SA-AKI). However, the structure, development, and thematic evolution of the SA-AKI–ferroptosis literature have not been clearly characterized. This study aimed to map the publication landscape, collaboration patterns, knowledge base, and emerging research themes in this field using bibliometric and science-mapping approaches.

**Methods:**

The Web of Science Core Collection (WoSCC) was used as the primary bibliometric database, and PubMed was used as an independent sensitivity check. A revised search strategy linked an SA-AKI concept block with a ferroptosis concept block using the Boolean operator AND, without using GPX4, ACSL4, SLC7A11, or other molecular markers as stand-alone search alternatives. Articles and reviews published from 2016 to 2025 were screened for substantive relevance to both SA-AKI and ferroptosis. The final WoSCC corpus was analyzed using VOSviewer 1.6.21 and CiteSpace 6.4. R2 for publication trends, collaboration networks, keyword co-occurrence, source co-citation, reference co-citation, clustering, timeline visualization, and burst detection.

**Results:**

Ninety-four WoSCC articles and reviews were assessed for eligibility, of which 68 were included in the final bibliometric corpus. Eligible publications first appeared in 2021 and increased from 2 publications in 2021 to 32 in 2025, with 50 of 68 publications (73.5%) published during 2024–2025. China contributed to 64 of 68 publications, whereas international collaboration remained limited. After keyword harmonization, 23 keywords entered the co-occurrence analysis; the most frequent were “ferroptosis,” “acute kidney injury,” “sepsis,” and “sepsis-associated acute kidney injury,” with Nrf2, GPX4, lipid peroxidation, oxidative stress, and related regulatory processes also represented. The reference co-citation network comprised 203 nodes and 858 links and formed 9 clusters (modularity Q = 0.5721; weighted mean silhouette S = 0.8355), connecting the clinical and pathophysiologic literature on SA-AKI with foundational ferroptosis biology and more recent SA-AKI-specific mechanistic studies. The co-cited-source network included 43 sources and 903 links. No sustained reference or keyword bursts were detected using a minimum duration of 2 years; only short 2024–2025 signals emerged in sensitivity analyses using a 1-year minimum duration.

**Conclusion:**

Research on ferroptosis in SA-AKI represents a relatively young and rapidly expanding literature that is highly concentrated geographically and characterized by limited international collaboration. Current knowledge structures link established ferroptosis biology with SA-AKI-specific experimental studies, while recent keyword patterns emphasize oxidative stress, lipid peroxidation, Nrf2/GPX4-related regulation, and related cellular processes. The limited duration of citation bursts and the predominance of preclinical literature indicate that the field remains in an early stage of development, and bibliometric signals should not be interpreted as evidence of established clinical translation.

## 1 Introduction

Sepsis is a life-threatening syndrome caused by a dysregulated host response to infection and is frequently complicated by multiple-organ dysfunction. Acute kidney injury (AKI) is among its most common and clinically important complications and is associated with adverse outcomes (Peerapornratana et al., 2019; Poston and Koyner, 2019; Zarbock et al., 2023; Zarbock et al., 2024). The pathophysiology of sepsis-associated acute kidney injury (SA-AKI) is complex and remains incompletely understood (Morrell et al., 2014; Zarbock et al., 2014; Peerapornratana et al., 2020; Ronco et al., 2019). It involves alterations in renal perfusion, dysregulated inflammatory and immune responses, and direct cellular injury (Bagshaw et al., 2007; Kellum et al., 2021). In recent years, increasing attention has focused on regulated cell-death pathways involved in septic kidney injury. Among these pathways, ferroptosis has attracted particular interest because of its close association with iron-dependent lipid peroxidation and oxidative injury (Dixon et al., 2012; Yang et al., 2014; Friedmann Angeli et al., 2014; Stockwell et al., 2017). These observations have motivated increasing investigation of ferroptosis-related mechanisms in SA-AKI.

Ferroptosis is a form of regulated cell death characterized by iron-dependent lipid peroxidation (Jiang et al., 2021; Tang et al., 2021; Li et al., 2020; Conrad and Pratt, 2019). Glutathione peroxidase 4 (GPX4), a major suppressor of lipid peroxidation, plays an important role in limiting ferroptotic injury (Seibt et al., 2019; Bersuker et al., 2019; Doll et al., 2019). Acyl-CoA synthetase long-chain family member 4 (ACSL4) can influence cellular susceptibility to ferroptosis by remodeling membrane lipid composition (Yang and Stockwell, 2016; Xie et al., 2016; Doll et al., 2017; Gaschler et al., 2017). Autophagy-dependent iron handling, particularly ferritinophagy, has also been implicated in the regulation of ferroptosis (Mancias et al., 2014; Gao et al., 2016; Hou et al., 2016). Accordingly, disturbances in iron homeostasis, lipid metabolism, and redox balance have become important mechanistic themes in studies of ferroptosis-associated kidney injury (Tang et al., 2019; Liu et al., 2022). However, the precise interactions among these processes in the context of SA-AKI remain incompletely defined (Ni et al., 2022; Feng et al., 2022).

Ferroptosis was formally defined in 2012 as a distinct form of regulated cell death characterized by iron-dependent accumulation of lipid peroxides (Dixon et al., 2012). It differs from apoptosis and other regulated cell-death processes in both its biochemical features and characteristic mitochondrial alterations (Dixon et al., 2012; Stockwell et al., 2017; Xie et al., 2016). Subsequent studies identified several major regulatory systems, including GPX4 and the cystine/glutamate antiporter system Xc−, of which SLC7A11 is a key component (Yang et al., 2014; Jiang et al., 2021; Seibt et al., 2019). These advances have established ferroptosis as an important biological process across multiple disease contexts, including malignancies, neurodegenerative disorders, and kidney diseases (Lin et al., 2022; Li et al., 2022).

Experimental studies have increasingly implicated ferroptosis in acute kidney injury (Chen et al., 2021; Guerrero-Hue et al., 2019; Liang et al., 2022). Reduced antioxidant capacity, altered GPX4 activity, impaired SLC7A11-dependent glutathione metabolism, and excessive lipid-peroxide accumulation have been investigated in experimental models of septic kidney injury (Xiao et al., 2021; Qiu et al., 2022; Qiongyue et al., 2022). Renal tubular epithelial cells appear particularly susceptible to ferroptosis-associated oxidative injury, and several studies have explored upstream regulatory pathways, including nuclear factor erythroid 2-related factor 2 (Nrf2), in this context (Guo et al., 2022; Won et al., 2024). These findings suggest that ferroptosis-related pathways may contribute to SA-AKI pathophysiology and represent active areas of preclinical investigation; however, their clinical relevance and therapeutic applicability remain to be established.

As interest in ferroptosis has expanded, its potential involvement in sepsis-related organ injury has also received increasing attention (Stockwell et al., 2017; Jiang et al., 2021; Tang et al., 2021; Li et al., 2020). Studies have examined ferroptosis in several sepsis-associated organ injuries, including kidney injury (Xiao et al., 2021; Qiu et al., 2022; Qiongyue et al., 2022), and recent SA-AKI investigations have explored regulatory pathways involving GPX4, Nrf2, SIRT1, and related oxidative-stress mechanisms (Guo et al., 2022; Won et al., 2024). Nevertheless, the SA-AKI-specific ferroptosis literature remains relatively specialized, and its overall publication structure, collaboration patterns, knowledge base, and thematic evolution have not been clearly mapped.

Traditional narrative reviews are useful for summarizing biological mechanisms but may not readily reveal the structural relationships, collaboration patterns, and temporal evolution of a rapidly developing research field. Bibliometric and science-mapping approaches provide complementary quantitative tools for examining publication output, co-authorship relationships, co-citation structures, keyword co-occurrence, and temporal trends (Donthu et al., 2021; van Eck and Waltman, 2010; Waltman et al., 2010; van Eck and Waltman, 2009). Such analyses can help characterize major contributors, the intellectual knowledge base, and evolving research themes within a defined literature corpus (Donthu et al., 2021; Waltman et al., 2010).

Therefore, this study aimed to characterize the research landscape of ferroptosis in SA-AKI during 2016–2025 using bibliometric and science-mapping approaches. The Web of Science Core Collection (WoSCC) was used as the primary bibliometric and citation-network database, while PubMed was used as an independent sensitivity check. We evaluated publication trends, geographic and collaborative patterns, leading sources and contributors, keyword co-occurrence, source and reference co-citation structures, and temporal thematic patterns. By focusing on a field-specific SA-AKI–ferroptosis corpus, this study sought to provide a reproducible overview of the current knowledge structure and evolving research themes without extending bibliometric signals beyond the evidence they directly support.

## 2 Materials and methods

### 2.1 Data acquisition

The Web of Science Core Collection (WoSCC) was used as the primary database for bibliometric and citation-network analyses, while PubMed was used as an independent sensitivity and coverage check. The final searches were conducted on 13 August 2026, and were restricted to final publication years from 2016 to 2025.

To specifically identify publications addressing ferroptosis in sepsis-associated acute kidney injury (SA-AKI), the WoSCC search strategy combined an SA-AKI concept block with a ferroptosis concept block using the Boolean operator AND. The final search strategy was:

TS=((“sepsis-associated acute kidney injury” OR “sepsis-induced acute kidney injury” OR “sepsis induced acute kidney injury” OR “septic acute kidney injury” OR “sepsis-associated AKI” OR “sepsis-induced AKI” OR “septic AKI” OR “SA-AKI” OR “S-AKI” OR ((sepsis OR septic) AND (“acute kidney injury” OR “acute renal injury” OR AKI))) AND (ferropt* OR “iron-dependent cell death” OR “iron dependent cell death”)) AND (FPY=(2016–2020) OR FPY=(2021–2025)).

GPX4, ACSL4, SLC7A11, and other ferroptosis-related molecular markers were not used as stand-alone OR terms. After restriction to Articles and Reviews, 94 candidate WoSCC records were identified. Records were exported in plain-text format as Full Record and Cited References, including bibliographic information on authors, affiliations, source journals, publication year, document type, keywords, cited references, DOI, WoS accession number, and citation counts.

PubMed was searched using the following sensitivity-check strategy:

((“Sepsis” [Mesh] OR sepsis [tiab] OR septic [tiab] OR “sepsis-associated acute kidney injury” [tiab] OR “sepsis-induced acute kidney injury” [tiab] OR “sepsis induced acute kidney injury” [tiab] OR “septic acute kidney injury” [tiab] OR “sepsis-associated AKI” [tiab] OR “sepsis-induced AKI” [tiab] OR “septic AKI” [tiab] OR “SA-AKI” [tiab] OR “S-AKI” [tiab]) AND (“Acute Kidney Injury” [Mesh] OR “acute kidney injury” [tiab] OR “acute renal injury” [tiab] OR AKI [tiab]) AND (“Ferroptosis” [Mesh] OR ferropt* [tiab] OR “iron-dependent cell death” [tiab] OR “iron dependent cell death” [tiab])) AND 2016/01/01:2025/12/31 [dp] AND (“Journal Article” [pt] OR Review [pt]).

PubMed records were exported with PMID, DOI, title, publication year, publication type, and abstract. Records were matched to the WoSCC candidate set using DOI as the primary identifier, followed by PMID and normalized title matching when necessary. PubMed-only records were screened using the same eligibility criteria but were not merged into the WoSCC citation or co-citation networks. Because PubMed publication-date filters may include electronic publication dates that differ from the final journal issue year, PubMed-only records were additionally checked against their final publication year.

### 2.2 Inclusion and exclusion criteria

Publications were considered eligible if they met all of the following criteria: (1) the document type was an Article or Review; (2) the final publication year was between 2016 and 2025; and (3) the publication substantively addressed all three components of the study topic: sepsis or septic illness, acute kidney injury or renal injury, and ferroptosis. Experimental studies were included when ferroptosis was directly investigated in an SA-AKI or sepsis-related renal injury model. Review articles were included only when the intersection between SA-AKI and ferroptosis constituted a substantive component of the review rather than a peripheral example.

Records were excluded for the following reasons (E1) AKI or renal injury not associated with sepsis; (E2) sepsis without substantive AKI or renal-injury content; (E3) sepsis and AKI were both addressed, but ferroptosis was only mentioned incidentally; (E4) broad reviews in which the SA-AKI–ferroptosis intersection was peripheral (E5) duplicate records; or (E6) other prespecified reasons documented during screening.

Two reviewers independently assessed the 94 candidate WoSCC records. Disagreements were resolved by discussion and consensus, with full-text assessment performed for borderline records when necessary. After consensus screening, 68 publications were retained in the final WoSCC bibliometric corpus. The screening procedure and reasons for exclusion are summarized in Figure 1.

**FIGURE 1:**
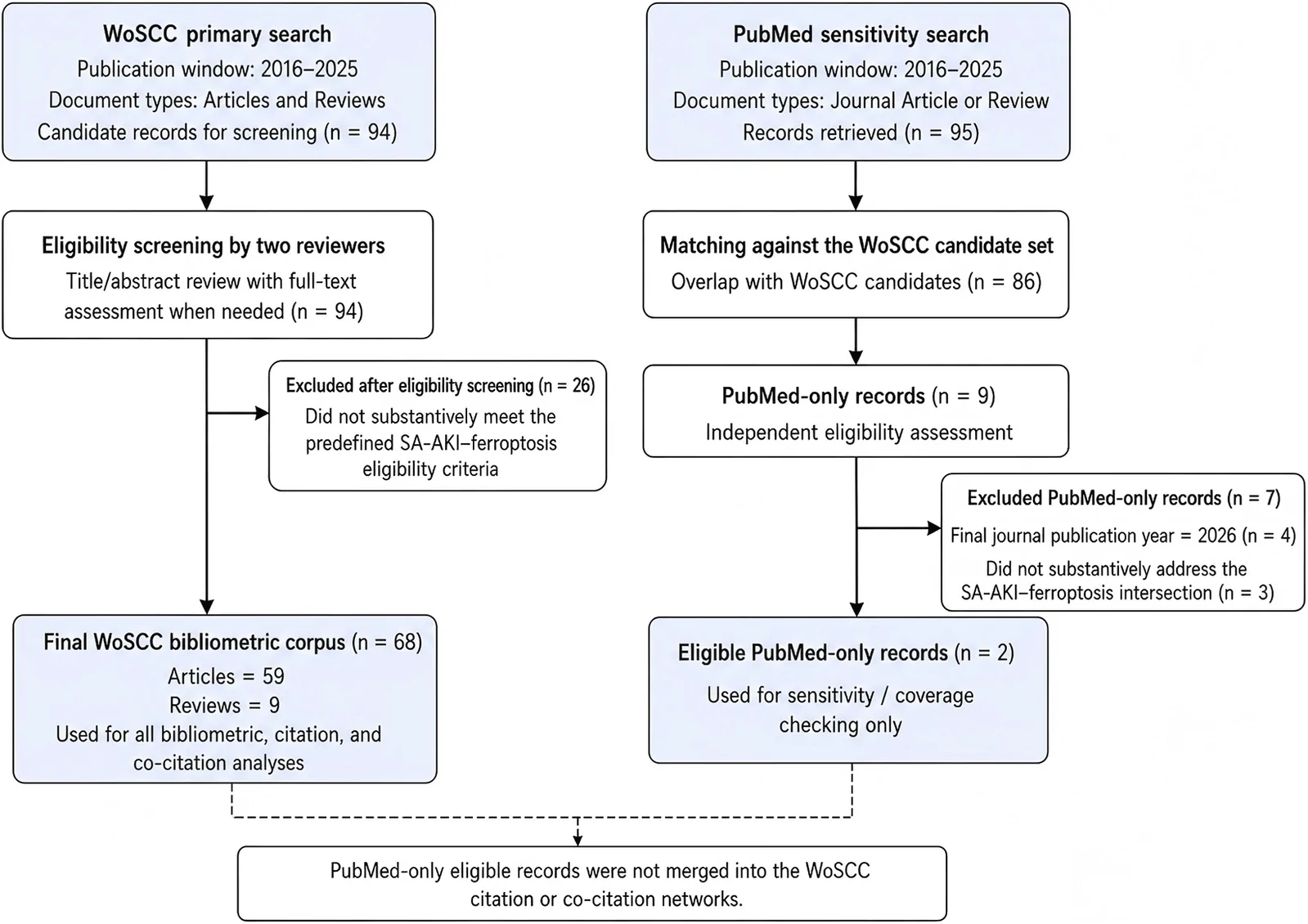
Flowchart of literature search.

### 2.3 Data analysis and visualization

Bibliographic information was extracted from the final 68-record WoSCC corpus, including publication year, document type, authors, affiliations, countries/regions, source journals, author keywords, cited references, and WoS citation counts. Before network analysis, obvious variants in keywords and organization names were harmonized. A predefined VOSviewer thesaurus was used for keyword cleaning. For example, “SA-AKI”, “S-AKI”, and “sepsis-induced AKI” were harmonized to “sepsis-associated acute kidney injury”, “AKI” to “acute kidney injury”, “LPS” to “lipopolysaccharide”, and “glutathione peroxidase 4” to “GPX4”. Ambiguous author names represented only by surname and initials were interpreted cautiously and were not assumed to correspond to a unique individual without sufficient evidence.

VOSviewer version 1.6.21 was used for co-authorship, keyword co-occurrence, and source co-citation analyses (van Eck and Waltman, 2010; Waltman et al., 2010; van Eck and Waltman, 2009). For keyword co-occurrence analysis, full counting and association-strength normalization were used. A minimum occurrence threshold of 2 was applied, resulting in 23 cleaned keywords in the final network. Node size represented keyword occurrence frequency, link strength reflected co-occurrence strength, and colors indicated network clusters; the overlay visualization represented the average publication year of each keyword.

For institutional co-authorship analysis, full counting was used and institutions with at least two publications were included in the displayed network. For author co-authorship analysis, fractional counting was used to reduce the influence of publications with large author teams; authors with at least two publications were retained in the displayed network. These collaboration analyses were interpreted descriptively because of the small and geographically concentrated final corpus.

Source co-citation analysis was performed using fractional counting, with a minimum threshold of 20 co-citations per cited source. Of 798 cited sources, 43 met the threshold and were included in the final co-citation network. Co-cited-author analysis was also performed using fractional counting, with a minimum threshold of 10 co-citations per cited author; 40 cited authors met this criterion. Because cited-author records were represented primarily by surname and initials, potentially ambiguous names were interpreted cautiously.

CiteSpace version 6.4. R2 Basic was used for reference co-citation, clustering, timeline, keyword, and citation-burst analyses (Chen, 2006). The analysis period was set to 2016–2025 with a time slice of 1 year. Because no eligible records were identified before 2021, the effective non-empty time slices were 2021–2025. For reference co-citation analysis, the node type was Cited Reference, node selection was based on the g-index with k = 25, and cosine similarity was used to measure link strength within slices. Pathfinder pruning and pruning of sliced networks were enabled, whereas minimum spanning tree pruning and pruning of the merged network were disabled. The LRF, L/N, and LBY parameters were set to −1. Cluster labels were initially generated using the log-likelihood ratio (LLR) algorithm.

The final Pathfinder reference co-citation network contained 203 nodes and 858 links, with a modularity Q of 0.5721 and a weighted mean silhouette value of 0.8355. The CiteSpace keyword network contained 122 nodes and 444 links, with Q = 0.5499 and S = 0.8234. Because several automatically generated LLR cluster labels were overly generic or too narrow, cluster numbers were retained as the primary identifiers, while short descriptive themes were assigned after reviewing representative highly co-cited references within each cluster. These themes were used only to facilitate bibliometric interpretation and were not treated as direct evidence of biological mechanisms.

Citation-burst detection was initially performed using a minimum burst duration of 2 years. No reference or keyword bursts met this criterion. A sensitivity analysis was therefore conducted by changing only the minimum duration from 2 to 1 year. This sensitivity analysis identified three reference bursts and one keyword burst, all occurring during 2024–2025. The default and sensitivity results were reported separately to avoid overinterpretation of short-lived signals.

Country-level publication output was calculated using full counting, whereby a multinational publication contributed one publication to each participating country. To reduce bias arising from different citation-exposure periods and document types, a normalized citation score (NCS) was calculated for each publication as its WoS citation count divided by the mean citation count of publications from the same publication year and document type (Article or Review). A country-level mean normalized citation score (MNCS) was then calculated as the mean NCS of publications attributed to that country. Because six of the seven contributing countries were represented by only one publication, these citation indicators were interpreted descriptively and were not used to rank national research quality.

Network structures and cluster assignments were generated using VOSviewer and CiteSpace. For publication-quality presentation, the final exported map, network, and JSON files were reformatted programmatically using Python while retaining the original node coordinates, cluster assignments, and link weights. No bibliometric network was recalculated or altered during graphical reformatting.

## 3 Results

After restriction to Articles and Reviews, 94 WoSCC records were assessed for eligibility, including 72 Articles and 22 Reviews. Following independent screening and consensus assessment, 68 publications were retained in the final WoSCC bibliometric corpus, comprising 59 Articles and 9 Reviews. The PubMed sensitivity search retrieved 95 records, of which 86 overlapped with the 94 WoSCC candidates, leaving 9 PubMed-only records. After assessment of topic eligibility and final journal publication year, 2 PubMed-only records met the predefined 2016–2025 eligibility criteria, whereas 7 were excluded, including 4 with a final journal publication year of 2026 and 3 that did not substantively address the SA-AKI–ferroptosis intersection. PubMed-only records were not incorporated into the WoSCC citation or co-citation networks. The literature-selection process is summarized in Figure 1.

### 3.1 Annual publication trends and country/region contributions

No eligible publications were identified between 2016 and 2020. The first eligible publications appeared in 2021, followed by a marked increase in output during the subsequent years. The annual numbers of publications were 2 in 2021, 9 in 2022, 7 in 2023, 18 in 2024, and 32 in 2025 (Figure 2A). Of the 68 included publications, 50 (73.5%) were published during 2024–2025, including 32 (47.1%) in 2025 alone. These findings indicate recent acceleration in a relatively young research field rather than the decade-long S-shaped growth pattern suggested by the broader initial search.

**FIGURE 2:**
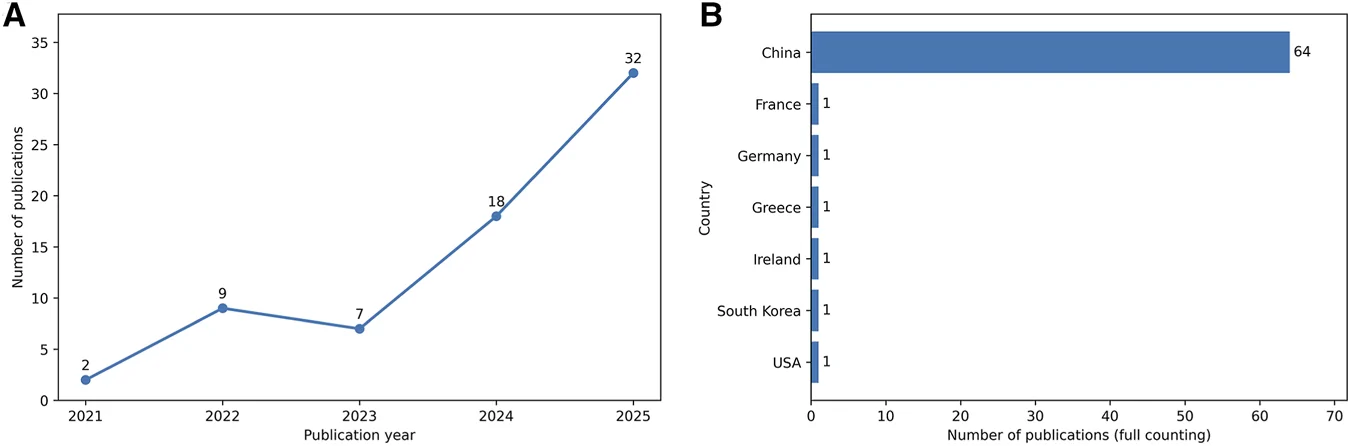
Temporal and geographic characteristics of the eligible literature. **(A)** Annual number of eligible publications. Although the search window covered 2016–2025, the final corpus contained no eligible records from 2016 to 2020; the 68 included publications were published from 2021 to 2025. **(B)** Country-level publication output using full counting. A multinational publication was counted once for each contributing country; therefore, the summed country counts can exceed the total number of publications.

Seven countries were represented in the final corpus. China contributed to 64 of the 68 publications (94.1% using full counting), while France, Germany, Greece, Ireland, South Korea, and the United States each contributed one publication (Figure 2B; Table 1). Only two publications involved international co-authorship, indicating that cross-country collaboration remained limited.

**TABLE 1:** Geographic distribution and normalized citation profile of the final WoS corpus.

Country	Publications	Share	Total citations	Mean citations	MNCS	International coauthored
China	64	94.1%	1544	24.1	1.021	2
France	1	1.5%	0	0.0	0.000	0
Germany	1	1.5%	176	176.0	1.000	0
Greece	1	1.5%	62	62.0	1.305	0
Ireland	1	1.5%	23	23.0	1.260	1
South Korea	1	1.5%	9	9.0	0.374	0
USA	1	1.5%	0	0.0	0.000	1

MNCS is the mean of paper-level citation scores normalized within the same publication year and document type. Because all non-China countries were represented by one publication, country-level citation metrics are descriptive and should not be interpreted as research-quality rankings.

To account for differences in citation exposure, citation counts were normalized by publication year and document type. China had a mean normalized citation score (MNCS) of approximately 1.02, close to the corpus reference level. Because each of the other six countries was represented by only one publication, their normalized citation values were considered descriptive and were not used for national research-quality comparisons.

### 3.2 Institutional contributions and leading publication venues

Institutional output was highly concentrated. Wuhan University was the most productive institution among those meeting the mapping threshold, contributing 7 publications. Southern Medical University, Sun Yat-sen University, Central South University, and Harbin Medical University each contributed 3 publications, while several additional institutions contributed 2 publications. The institutional collaboration network was relatively sparse, with most links occurring among Chinese institutions and limited cross-border institutional connectivity (Supplementary Figure S1; Supplementary Table S1).

The 68 publications were distributed across a relatively broad range of journals. International Immunopharmacology was the most productive source journal, with 6 publications. Nine additional journals contributed 2 publications each, including Renal Failure, Redox Report, Frontiers in Immunology, Free Radical Biology and Medicine, Cell Death Discovery, Translational Andrology and Urology, Kaohsiung Journal of Medical Sciences, Shock, and Journal of Molecular Histology (Table 2).

**TABLE 2:** Leading journals by number of included publications.

Journal	Documents	Share	Total citations	Mean citations	Mean publication year
International Immunopharmacology	6	8.8%	231	38.5	2023.50
Renal Failure	2	2.9%	72	36.0	2023.00
Redox Report	2	2.9%	70	35.0	2024.00
Frontiers In Immunology	2	2.9%	54	27.0	2024.50
Free Radical Biology And Medicine	2	2.9%	44	22.0	2024.50
Cell Death Discovery	2	2.9%	36	18.0	2024.50
Translational Andrology And Urology	2	2.9%	24	12.0	2024.00
Kaohsiung Journal Of Medical Sciences	2	2.9%	11	5.5	2024.50
Shock	2	2.9%	7	3.5	2025.00
Journal of Molecular Histology	2	2.9%	3	1.5	2025.00

The source co-citation analysis identified 798 cited sources, of which 43 met the threshold of at least 20 co-citations. These 43 sources formed a dense network containing 903 links (Figure 3A). The most frequently co-cited sources were Cell (110 co-citations), Cell Death & Disease (104), Free Radical Biology and Medicine (92), Redox Biology (88), Nature (88), Kidney International (81), Journal of the American Society of Nephrology (77), and Frontiers in Pharmacology (76). The 15 most frequently co-cited sources are shown in Figure 3B, with detailed network characteristics provided in Supplementary Table S3.

**FIGURE 3:**
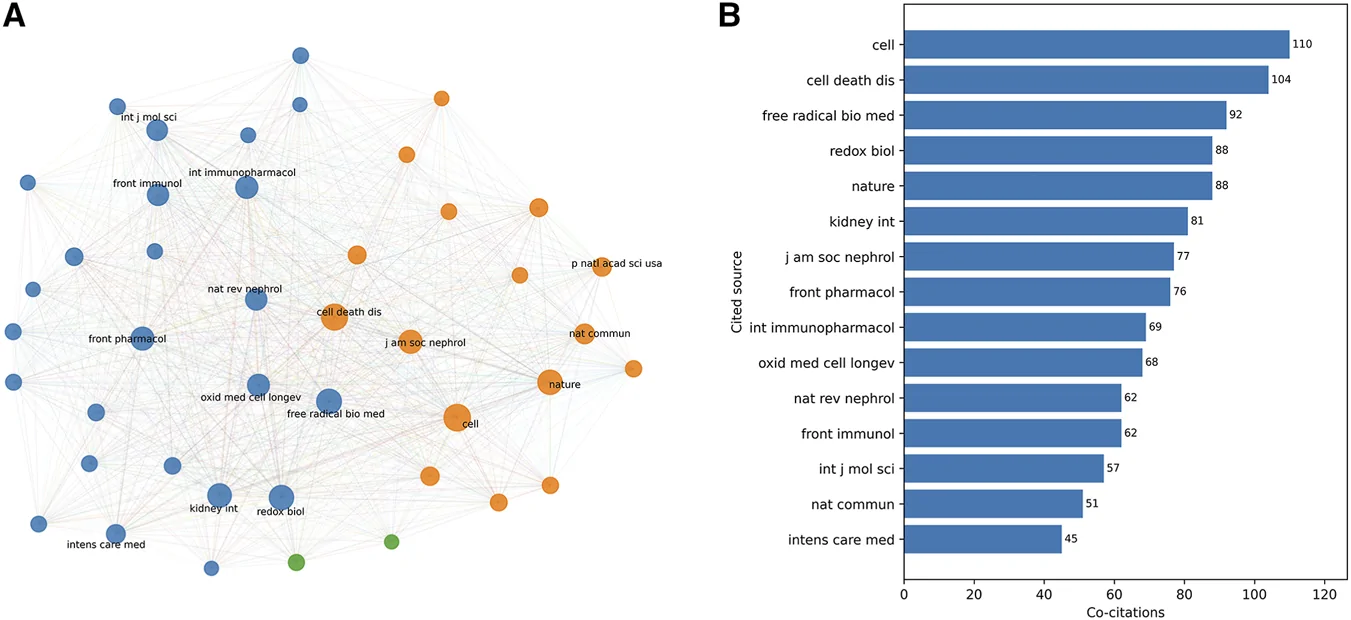
Co‐cited source network of the final corpus. Co‐citation analysis of cited sources was conducted in VOSviewer using fractional counting and a minimum threshold of 20 citations per source. Forty‐three cited sources met the threshold, forming 903 links across 3 clusters (total link strength = 927.48). **(A)** Network visualization of journal co‐citation clusters; node size is proportional to co‐citation frequency, link thickness reflects co‐citation link strength, and node grouping denotes VOSviewer cluster membership. **(B)** Horizontal bar chart showing journals ranked by co‐citation frequency.

### 3.3 Authors, co-cited authors and collaboration patterns

Author-level productivity was dispersed across the final corpus. Among authors meeting the VOSviewer threshold of at least two publications, no individual author contributed more than two publications, indicating the absence of a single highly dominant author group within the field-specific dataset. The author co-authorship network was correspondingly sparse and fragmented into several small collaborative groups (Supplementary Figure S2; Supplementary Table S2).

In contrast, the co-cited-author network showed a more interconnected intellectual structure. Dixon SJ was the most frequently co-cited author, with 46 co-citations, followed by Wang Y (29), Stockwell BR (27), Doll S (26), Yang WS (26), Peerapornratana S (25), Chen X (24), and Poston JT (23) (Supplementary Figure S3; Supplementary Table S4). However, because cited-author records were exported primarily in surname-plus-initial format, abbreviated names such as “Wang Y″ and “Chen X″ may represent more than one individual and were therefore interpreted cautiously.

### 3.4 Reference co-citation structure, timeline, and citation bursts

The Pathfinder reference co-citation network contained 203 cited references and 858 links and was partitioned into 9 clusters (Figure 4A). The network showed moderate structural separation and good within-cluster consistency, with a modularity Q of 0.5721 and a weighted mean silhouette value of 0.8355.

**FIGURE 4:**
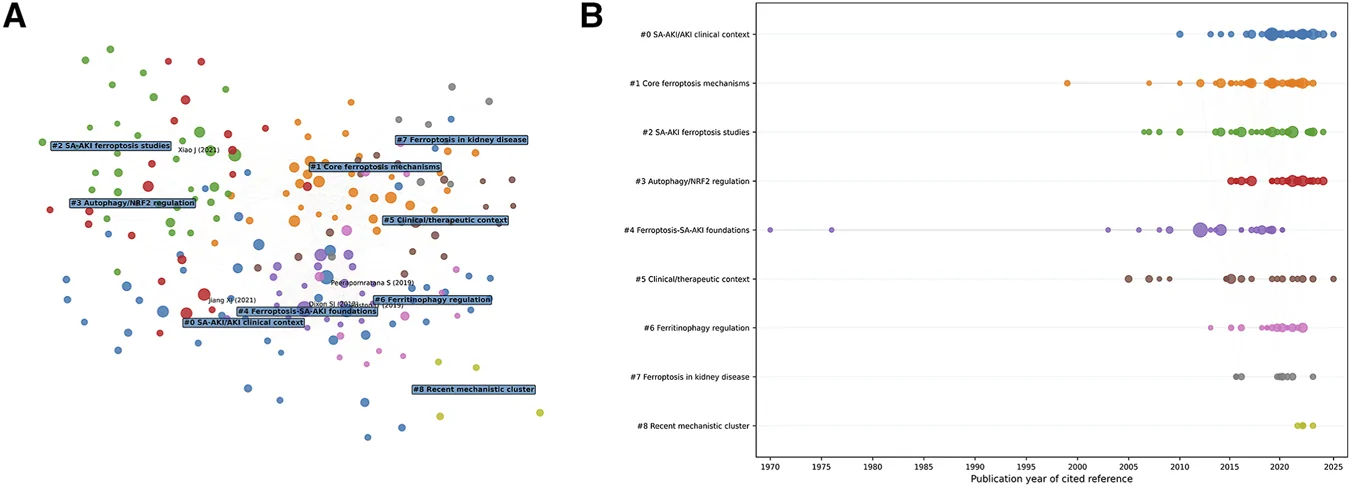
Reference co-citation structure and timeline of the knowledge base. CiteSpace reference co-citation analysis was performed using 1-year time slices, g-index selection (k = 25), cosine link strength, within-slice links, Pathfinder pruning, and pruning of sliced networks. **(A)** The pruned reference co-citation network contained 203 nodes, 858 links, and 9 clusters (modularity Q = 0.5721; weighted mean silhouette S = 0.8355). Node size in the reformatted figure is proportional to local co-citation frequency in the CiteSpace export. **(B)** Timeline visualization of the same co-citation structure. The publication years on the horizontal axis refer to cited references and can therefore precede the 2021–2025 publication years of the eligible source articles. Automatic LLR cluster labels were interpreted cautiously; where labels were overly generic or narrow, the manuscript text describes cluster themes conservatively on the basis of their member references.

The most frequently co-cited reference was Dixon et al. (2012) (2012, Cell), with 33 local co-citations, followed by Peerapornratana et al. (2019) (2019, Kidney International; 24), Poston and Koyner (2019) (2019, BMJ; 22), Xiao et al. (2021) (2021, Cell Bioscience; 18), and Jiang et al. (2021) (2021, Nature Reviews Molecular Cell Biology; 17) (Table 3). Other highly co-cited references included Friedmann Angeli et al. (2014), Zarbock et al. (2023), Qiongyue et al. (2022), Bersuker et al. (2019), and Doll et al. (2019).

**TABLE 3:** Most frequently co-cited references in the reference co-citation network.

References	Local co-citations	Betweenness centrality	Cluster
Dixon SJ, 2012, CELL, V149, P1060, DOI 10.1016/j.cell.2012.03.042	33	0.142	4
Peerapornratana S, 2019, KIDNEY INT, V96, P1083, DOI 10.1016/j.kint.2019.05.026	24	0.166	0
Poston JT, 2019, BMJ-BRIT MED J, V364, P0, DOI 10.1136/bmj.k4891	22	0.066	0
Xiao J, 2021, CELL BIOSCI, V11, P0, DOI 10.1186/s13578-021-00734-x	18	0.066	2
Jiang XJ, 2021, NAT REV MOL CELL BIO, V22, P266, DOI 10.1038/s41580-020-00324-8	17	0.037	3
Angeli JPF, 2014, NAT CELL BIOL, V16, P1180, DOI 10.1038/ncb3064	16	0.274	4
Zarbock A, 2023, NAT REV NEPHROL, V19, P401, DOI 10.1038/s41581-023-00683-3	14	0.029	0
Qiongyue Z, 2022, FRONT PHARMACOL, V13, P0, DOI 10.3389/fphar.2022.857067	14	0.004	3
Bersuker K, 2019, NATURE, V575, P688, DOI 10.1038/s41586-019-1705-2	13	0.153	1
Doll S, 2019, NATURE, V575, P693, DOI 10.1038/s41586-019-1707-0	13	0.148	1

Cluster numbers refer to the final CiteSpace Pathfinder reference co-citation network (203 nodes, 858 links, 9 clusters). Automatic LLR, labels should not be treated as mechanistic proof.

Inspection of representative highly co-cited references indicated that the 9 clusters broadly connected several components of the field’s knowledge base, including the clinical and pathophysiologic context of SA-AKI/AKI, foundational ferroptosis mechanisms, SA-AKI-specific ferroptosis studies, autophagy/NRF2-related regulation, ferritinophagy-related regulation, and ferroptosis in kidney disease. The cluster descriptors shown in Figure 4 were assigned conservatively after reviewing representative cluster members and were used as bibliometric summaries rather than mechanistic classifications.

The reference timeline demonstrated that the SA-AKI–ferroptosis literature draws on a substantially older knowledge base (Figure 4B). Foundational references on AKI, sepsis, oxidative injury, and ferroptosis predated the first eligible field-specific publications, whereas the included SA-AKI–ferroptosis corpus itself was concentrated in 2021–2025. Therefore, the presence of older cited references should not be interpreted as evidence that field-specific eligible publications existed before 2021.

No reference bursts met the default minimum burst-duration criterion of 2 years. In the sensitivity analysis in which only the minimum duration was reduced from 2 to 1 year, three reference bursts were detected, all extending from 2024 to 2025. These involved Tan CY (2021, International Journal of Molecular Medicine; burst strength approximately 2.31), Li JR (2023, European Journal of Pharmacology; approximately 1.93), and Qiongyue Z (2022, Frontiers in Pharmacology; approximately 1.92). Because these signals were short-lived and emerged only under the sensitivity setting, they were treated as supplementary indicators of recent citation activity rather than stable long-term research fronts.

### 3.5 Keyword co-occurrence and temporal evolution

After thesaurus-based harmonization, 23 keywords meeting the minimum occurrence threshold of 2 were included in the VOSviewer co-occurrence network (Figure 5A; Table 4). The most frequent keyword was “ferroptosis” (53 occurrences), followed by “acute kidney injury” (36), “sepsis” (23), and “sepsis-associated acute kidney injury” (23). Other recurrent terms included Nrf2 (7), GPX4 (6), lipopolysaccharide (5), inflammation (4), lipid peroxidation (4), m6A (4), and oxidative stress (4).

**FIGURE 5:**
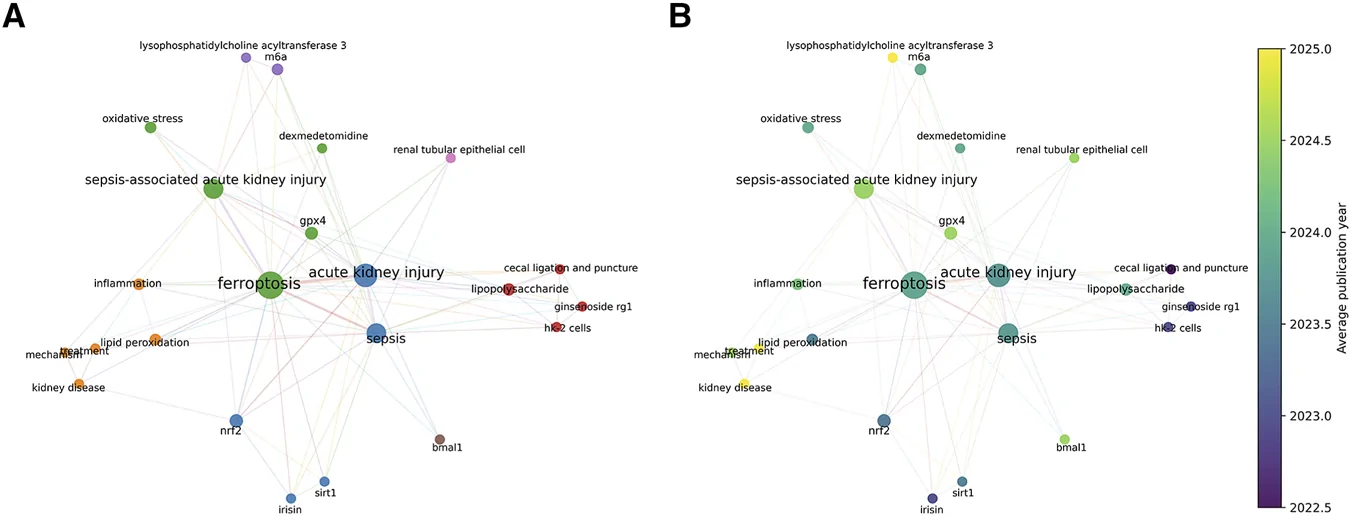
Keyword co-occurrence structure and temporal overlay in the final corpus. After thesaurus-based harmonization, 23 keywords meeting the minimum occurrence threshold of 2 were included. **(A)** Keyword co-occurrence network. Node size is proportional to keyword occurrence, link thickness reflects co-occurrence strength, and node grouping denotes VOSviewer cluster membership. **(B)** Overlay visualization of the same network, in which node position and size are unchanged and node color represents the average publication year of publications containing each keyword.

**TABLE 4:** High-frequency cleaned keywords.

Keyword	Occurrences	Total link strength	Mean publication year	VOS cluster
Ferroptosis	53	116.0	2023.91	3
Acute kidney injury	36	88.0	2023.72	1
Sepsis	23	64.0	2023.78	1
Sepsis-associated acute kidney injury	23	33.0	2024.48	3
nrf2	7	22.0	2023.43	1
gpx4	6	15.0	2024.50	3
Lipopolysaccharide	5	16.0	2024.00	4
Inflammation	4	11.0	2024.25	2
Lipid peroxidation	4	10.0	2023.50	2
m6a	4	8.0	2024.00	5
Oxidative stress	4	8.0	2024.00	3
Ginsenoside rg1	2	10.0	2023.00	4
Hk-2 cells	2	10.0	2023.00	4
Cecal ligation and puncture	2	9.0	2022.50	4
sirt1	2	8.0	2023.50	1

Keywords were harmonized using the prespecified thesaurus before network construction. The table reports the 15 most frequent of the 23 keywords entering the final map.

The keyword overlay map provided a temporal view of thematic emphasis within the short 2021–2025 study period (Figure 5B). The average publication years were approximately 2023.91 for “ferroptosis”, 2023.72 for “acute kidney injury”, 2023.78 for “sepsis”, 2024.48 for “sepsis-associated acute kidney injury”, and 2024.50 for “GPX4”. Several mechanistic terms, including inflammation, oxidative stress, m6A, GPX4, and SA-AKI-specific terminology, therefore appeared relatively later within the observation period. Given the narrow five-year span, however, these differences were interpreted as changes in thematic emphasis rather than evidence of discrete chronological research stages.

Under the default minimum burst-duration criterion of 2 years, no keyword burst was detected. When the minimum duration was reduced to 1 year in the sensitivity analysis, only “AKI” showed a burst signal (strength approximately 1.86) during 2024–2025. The limited number and duration of keyword bursts suggest that sustained research-front signals have not yet become firmly established in this young literature.

## 4 Discussion

This study provides a focused bibliometric and science-mapping analysis of the literature specifically addressing ferroptosis in sepsis-associated acute kidney injury (SA-AKI). In contrast to the much broader literature set obtained using the original search strategy, the revised disease-specific corpus contained 68 eligible publications, all published between 2021 and 2025. Publication activity increased substantially in the most recent years, with 50 of the 68 publications (73.5%) appearing during 2024–2025. These findings suggest that SA-AKI–ferroptosis research is a relatively young and rapidly expanding field rather than a mature research domain with a decade-long developmental trajectory. Accordingly, the present results are better interpreted as a snapshot of an emerging specialty whose intellectual and collaborative structures are still being formed.

The geographic distribution of publications was highly concentrated. China contributed to 64 of the 68 publications, while only six other countries were represented and each contributed one publication. Moreover, only two publications involved international co-authorship. This pattern indicates that the recent expansion of SA-AKI–ferroptosis research has occurred predominantly within a geographically concentrated research community, with limited cross-country collaboration. Importantly, publication volume should not be equated with research quality or scientific influence. After normalization for publication year and document type, the MNCS for China was approximately 1.02, close to the corpus reference level. Because each of the other countries was represented by only a single publication, their normalized citation values were statistically unstable and do not support meaningful national quality rankings. The revised analysis therefore does not reproduce the previous interpretation that differences in raw citation counts reflect differences in national research quality.

The co-citation analyses provide a clearer view of the intellectual foundations on which this emerging literature is built. The most frequently co-cited sources included Cell, Cell Death & Disease, Free Radical Biology and Medicine, Redox Biology, Nature, Kidney International, and the Journal of the American Society of Nephrology. At the reference level, highly co-cited publications included the foundational description of ferroptosis by Dixon et al. (Dixon et al., 2012), major reviews and mechanistic studies of ferroptosis (Stockwell et al., 2017; Jiang et al., 2021; Tang et al., 2021), and key clinical and pathophysiological literature concerning AKI and sepsis (Peerapornratana et al., 2019; Poston and Koyner, 2019; Zarbock et al., 2023). Thus, the knowledge base of SA-AKI–ferroptosis research is not composed solely of SA-AKI-specific studies. Rather, it integrates established ferroptosis biology with the broader AKI and sepsis literature, upon which a smaller body of disease-specific experimental studies has subsequently developed. This distinction is important because highly co-cited foundational ferroptosis papers provide conceptual support for the field but should not be interpreted as direct clinical evidence in SA-AKI.

The reference co-citation network further supported this mixed intellectual structure. The Pathfinder network contained 203 nodes and 858 links and showed satisfactory clustering characteristics (Q = 0.5721; S = 0.8355). Inspection of representative references within the nine clusters indicated several broad themes, including the clinical and pathophysiological context of SA-AKI and AKI, core ferroptosis mechanisms, SA-AKI-specific ferroptosis studies, autophagy- and Nrf2-related regulation, ferritinophagy-associated processes, and ferroptosis in kidney disease. These themes are broadly consistent with the biological development of the field. Recent experimental studies have further investigated Nrf2/GPX4-centered antioxidant regulation in SA-AKI (Zan et al., 2024; Yang S. et al., 2024; Zheng et al., 2024). Autophagy- and lipid-metabolism-related regulation of ferroptosis has also emerged as a mechanistic theme (Yang Y. et al., 2024). Nevertheless, the cluster descriptors should be interpreted as summaries of co-citation structure rather than as evidence that any single pathway has been established as the dominant mechanism or a validated therapeutic target in patients.

Keyword analysis provided a complementary view of the active research themes. After thesaurus-based harmonization, the dominant terms were “ferroptosis,” “acute kidney injury,” “sepsis,” and “sepsis-associated acute kidney injury,” confirming that the revised corpus was tightly centered on the intended research question. Mechanistic keywords such as Nrf2, GPX4, oxidative stress, lipid peroxidation, inflammation, lipopolysaccharide, and m6A were also recurrent. These findings are biologically plausible in the context of experimental SA-AKI. GPX4 is a major suppressor of lipid peroxidation, while Nrf2-related antioxidant responses and changes in glutathione metabolism have been repeatedly investigated in experimental kidney injury. Recent studies have also expanded the mechanistic spectrum to Nrf2/FSP1-related ferroptosis resistance (Zhang et al., 2024). In parallel, m6A modification, GPX4 autophagic degradation, and transcriptional regulation of Nrf2-associated pathways have been investigated in SA-AKI models (Xu et al., 2025; Wang et al., 2025; Zhang et al., 2025). Autophagy-associated regulation of GPX4 and ferroptosis has also been reported in renal injury models (Mancias et al., 2014; Gao et al., 2016; Hou et al., 2016) and in more recent SA-AKI studies (Yang Y. et al., 2024; Wang et al., 2025). However, keyword frequency is a measure of research attention rather than biological effect size, causal importance, or therapeutic efficacy. Therefore, the prominence of GPX4, Nrf2, or other ferroptosis-related terms should be interpreted as indicating active areas of investigation rather than a hierarchy of clinically validated therapeutic targets.

The temporal analyses also argue for a cautious interpretation of emerging research fronts. Although several SA-AKI-specific and mechanistic keywords had relatively recent average publication years, the entire eligible publication period covered only 5 years. Differences in average publication year within such a short interval cannot reliably establish a sequential transition from basic research to clinical translation. Consistent with this interpretation, no reference or keyword bursts persisted for at least 2 years under the default burst-detection criterion. Only three reference bursts and one keyword burst emerged when the minimum duration was reduced to 1 year, and all were confined to 2024–2025. These short signals indicate recent attention but do not demonstrate stable long-term fronts. This result differs substantially from the original analysis, which appeared to show sustained bursts extending to 2026 and was used to support a transition toward therapeutic translation. The revised evidence instead suggests that the thematic structure remains dynamic and that durable research fronts have not yet been firmly established.

The findings also have implications for future research. First, the marked geographic concentration and limited international co-authorship suggest that broader cross-regional collaboration would improve the diversity and external relevance of the evidence base. Second, although the corpus contains an increasing number of experimental studies involving ferroptosis-related pathways, most available evidence remains preclinical (Fan et al., 2025; Bao et al., 2025; Tang et al., 2025). Recent studies involving BMAL1 regulation and epigenetic control further illustrate the continuing diversification of mechanistic hypotheses in this field (Chen et al., 2025; Yang et al., 2025). Future work should therefore emphasize reproducible mechanistic validation across independent models, clarification of whether ferroptosis is a causal contributor rather than a secondary marker of severe cellular injury, and assessment of ferroptosis-related biomarkers and pathways in well-characterized human SA-AKI cohorts. Third, bibliometric prominence alone should not be used to prioritize therapeutic targets. Translation of pathways such as Nrf2/GPX4, SLC7A11-associated antioxidant regulation, autophagy, or ferritinophagy will require evidence from appropriately designed translational and clinical studies rather than extrapolation from publication frequency or network centrality.

Overall, the revised analysis depicts SA-AKI–ferroptosis research as an emerging, rapidly expanding, but still predominantly preclinical field. Its current knowledge structure links foundational ferroptosis biology with established AKI and sepsis concepts and a growing body of SA-AKI-specific mechanistic studies. The concentration of recent publications and the appearance of oxidative stress-, lipid peroxidation-, Nrf2/GPX4-, and autophagy-related themes demonstrate increasing experimental interest, but the limited international collaboration, short citation-burst duration, and absence of mature clinical evidence argue against describing the field as having already entered a phase of established therapeutic translation.

## 5 Limitations

Several limitations should be considered when interpreting the present findings. First, the primary bibliometric corpus was derived from the Web of Science Core Collection, while PubMed was used only as an independent sensitivity and coverage check. Other bibliographic databases, such as Scopus and Embase, were not included, and relevant publications indexed exclusively in those databases may therefore have been missed. In addition, the analysis was restricted to Articles and Reviews and did not include conference abstracts, dissertations, or other forms of grey literature. All 68 publications in the final WoSCC corpus were published in English, and relevant non-English literature not captured by the present search and indexing strategy may be underrepresented. Second, the final field-specific corpus was relatively small and geographically concentrated. Of the 68 included publications, 64 involved China, whereas each of the other six contributing countries was represented by only one publication. This distribution limits the stability of country-, institution-, and author-level comparisons and makes cross-country rankings particularly sensitive to individual publications. Accordingly, geographic and collaboration indicators in this study should be interpreted descriptively rather than as measures of national or institutional research quality. Third, citation-based indicators are influenced by the time elapsed since publication. Although country-level citation impact was normalized by publication year and document type, publications from 2025 had substantially less time to accumulate citations than earlier publications, and citation patterns may continue to change as the field develops. More generally, citation frequency, co-citation strength, and network centrality primarily reflect patterns of scholarly attention and intellectual connectivity and should not be interpreted as direct measures of experimental quality, causal importance, clinical relevance, or therapeutic efficacy. Fourth, data cleaning and science-mapping procedures inevitably involve methodological judgment. Keyword variants were harmonized using a predefined thesaurus, and several automatically generated CiteSpace cluster labels were considered overly broad or overly specific. We therefore retained the numerical cluster identifiers and assigned short descriptive themes only after reviewing representative highly co-cited references within each cluster. These descriptors facilitate interpretation of the co-citation structure but should not be regarded as formal biological classifications. Similarly, co-cited-author records were frequently represented by surname and initials, creating potential ambiguity for common author names. Finally, PubMed-only eligible records were not incorporated into the WoSCC citation and co-citation networks because PubMed does not provide an equivalent cited-reference structure for the bibliometric analyses performed here. PubMed was therefore used to evaluate search coverage rather than to create a merged citation corpus. The searches were completed on 13 August 2026, with the publication window restricted to 2016–2025; subsequently indexed or corrected records may alter some bibliometric indicators in future updates. These limitations should be considered when interpreting the present maps and temporal patterns.

## 6 Conclusion

This bibliometric and science-mapping analysis identified 68 WoSCC publications specifically addressing ferroptosis in SA-AKI during 2016–2025, with eligible publications appearing only from 2021 onward. Publication activity increased substantially in 2024–2025, indicating that this is a relatively young and rapidly expanding research field. The literature was highly geographically concentrated, with China contributing to 64 of the 68 publications and international collaboration remaining limited. Co-citation analyses showed that the intellectual structure of the field integrates foundational ferroptosis biology with established AKI and sepsis literature and a growing body of SA-AKI-specific experimental studies. Keyword mapping highlighted ferroptosis, AKI, sepsis, SA-AKI, Nrf2, GPX4, oxidative stress, lipid peroxidation, and related regulatory processes as recurrent themes. However, sustained citation-burst signals were absent under the default analysis, and the available literature remains predominantly preclinical. These findings support characterization of SA-AKI–ferroptosis research as an emerging rather than mature field. Broader international collaboration, independent mechanistic validation, and well-designed studies using human SA-AKI samples or cohorts will be needed before bibliometrically prominent pathways can be considered clinically established biomarkers or therapeutic targets.

## Data Availability

The original contributions presented in the study are included in the article/Supplementary Material, further inquiries can be directed to the corresponding authors.
