# Supplementary material for: Research landscape and emerging themes of ferroptosis in sepsis-associated acute kidney injury: a bibliometric and science-mapping analysis (2016–2025)
Source: Front Pharmacol. 2026 Sep 24;17:1942452. doi: 10.3389/fphar.2026.1942452 (PMC13646085; doi:10.3389/fphar.2026.1942452)
Supplement: Supplementary file 1 [file Supplementaryfile1.docx]

**Supplementary Tables**

**Supplementary Table S1. Leading institutions**

| **Institution** | **Documents** | **Citations** | **Mean publication year** | **Mean normalized citations** | **Links** |
| --- | --- | --- | --- | --- | --- |
| wuhan univ | 7 | 268 | 2024.29 | 1.721 | 0 |
| southern med univ | 3 | 131 | 2023.00 | 0.635 | 1 |
| sun yat sen univ | 3 | 108 | 2022.67 | 0.625 | 1 |
| cent south univ | 3 | 95 | 2024.00 | 1.184 | 1 |
| harbin med univ | 3 | 42 | 2024.00 | 0.474 | 0 |
| huazhong univ sci & technol | 2 | 132 | 2023.00 | 1.262 | 0 |
| wenzhou med univ | 2 | 106 | 2022.50 | 1.036 | 0 |
| shanghai jiao tong univ | 2 | 51 | 2023.50 | 0.892 | 2 |
| shanxi med univ | 2 | 42 | 2024.50 | 0.954 | 0 |
| nanjing med univ | 2 | 38 | 2023.50 | 1.119 | 1 |

**Note.** The collaboration network is sparse; institution-level comparisons should remain descriptive.

**Supplementary Table S2. Most productive authors meeting the network threshold**

| **Author** | **Documents** | **Citations** | **Mean publication year** | **Mean normalized citations** | **Total link strength** |
| --- | --- | --- | --- | --- | --- |
| guo, jun | 2 | 132 | 2023.00 | 1.262 | 0.00 |
| gao, chunyan | 2 | 42 | 2024.50 | 0.954 | 2.00 |
| liu, hongxuan | 2 | 42 | 2024.50 | 0.954 | 2.00 |
| yang, meixia | 2 | 42 | 2024.50 | 0.954 | 2.00 |
| wang, yue | 2 | 40 | 2024.50 | 0.750 | 1.00 |
| wang, yuchen | 2 | 39 | 2024.00 | 0.731 | 0.00 |
| wang, xuan | 2 | 37 | 2024.00 | 1.629 | 0.00 |
| luo, jun | 2 | 29 | 2025.00 | 2.148 | 0.00 |
| li, wei | 2 | 26 | 2024.50 | 1.096 | 0.00 |
| li, yunlong | 2 | 24 | 2024.00 | 0.373 | 2.00 |

**Note.** Only authors entering the final VOSviewer network are shown. Sparse coauthorship limits strong collaboration inferences.

**Supplementary Table S3. Most frequently co-cited sources**

| **Cited source** | **Co-citations** | **Total link strength** | **Cluster** |
| --- | --- | --- | --- |
| cell | 110 | 102.99 | 2 |
| cell death dis | 104 | 97.73 | 2 |
| free radical bio med | 92 | 88.01 | 1 |
| redox biol | 88 | 81.65 | 1 |
| nature | 88 | 81.03 | 2 |
| kidney int | 81 | 77.77 | 1 |
| j am soc nephrol | 77 | 73.77 | 2 |
| front pharmacol | 76 | 71.23 | 1 |
| int immunopharmacol | 69 | 66.39 | 1 |
| oxid med cell longev | 68 | 64.62 | 1 |
| nat rev nephrol | 62 | 60.53 | 1 |
| front immunol | 62 | 58.62 | 1 |
| int j mol sci | 57 | 53.86 | 1 |
| nat commun | 51 | 48.86 | 2 |
| intens care med | 45 | 43.76 | 1 |

**Note.** The full co-cited-source network contained 43 sources and 903 links.

**Supplementary Table S4. Most frequently co-cited authors**

| **Co-cited author** | **Co-citations** | **Total link strength** | **Cluster** |
| --- | --- | --- | --- |
| dixon, sj | 46 | 44.57 | 1 |
| wang, y | 29 | 27.40 | 1 |
| stockwell, br | 27 | 26.35 | 1 |
| doll, s | 26 | 25.12 | 1 |
| yang, ws | 26 | 24.66 | 1 |
| peerapornratana, s | 25 | 25.00 | 2 |
| chen, x | 24 | 23.09 | 4 |
| poston, jt | 23 | 22.00 | 2 |
| guo, j | 20 | 19.53 | 3 |
| linkermann, a | 20 | 18.71 | 1 |
| jiang, xj | 19 | 19.00 | 3 |
| xiao, j | 18 | 18.00 | 2 |
| angeli, jpf | 18 | 17.90 | 1 |
| martin-sanchez, d | 18 | 17.89 | 1 |
| zarbock, a | 17 | 17.00 | 2 |

**Note.** Surname-plus-initial labels can merge distinct individuals; abbreviated names should therefore be interpreted cautiously.

**Supplementary Table S5. Burst-detection sensitivity results**

| **Type** | **Item** | **Strength** | **Begin** | **End** |
| --- | --- | --- | --- | --- |
| Reference | Tan CY, 2021, Int J Mol Med | 2.31 | 2024 | 2025 |
| Reference | Li JR, 2023, Eur J Pharmacol | 1.93 | 2024 | 2025 |
| Reference | Qiongyue Z, 2022, Front Pharmacol | 1.92 | 2024 | 2025 |
| Keyword | AKI | 1.857 | 2024 | 2025 |

**Note.** The default minimum burst duration of 2 years yielded no reference or keyword bursts. The listed signals appeared only in the prespecified sensitivity analysis with minimum duration reduced to 1 year; no other burst parameter was changed.
